# Cdc42 overexpression induces hyperbranching in the developing mammary gland by enhancing cell migration

**DOI:** 10.1186/bcr3487

**Published:** 2013-09-30

**Authors:** Kristi Bray, Melissa Gillette, Jeanette Young, Elizabeth Loughran, Melissa Hwang, James Cooper Sears, Tracy Vargo-Gogola

**Affiliations:** 1 Department of Biological Sciences and the Harper Cancer Research Institute, University of Notre Dame, Notre Dame, IN 46556, USA; 2 Department of Biochemistry and Molecular Biology, Indiana University School of Medicine and the Simon Cancer Center, 1234 Notre Dame Ave, South Bend, IN 46617, USA

## Abstract

**Introduction:**

The Rho GTPase Cdc42 is overexpressed and hyperactivated in breast tumors compared to normal breast tissue. Cdc42 regulates key processes that are critical for mammary gland morphogenesis and become disrupted during the development, progression, and metastasis of breast cancer. However, the contribution of Cdc42 to normal and neoplastic mammary gland development *in vivo* remains poorly understood. We were therefore interested in investigating the effects of Cdc42 overexpression on mammary gland morphogenesis as a first step toward understanding how its overexpression may contribute to mammary tumorigenesis.

**Methods:**

We developed a tetracycline-regulatable Cdc42 overexpression mouse model in which Cdc42 can be inducibly overexpressed in the developing mammary gland. The effects of Cdc42 overexpression during postnatal mammary gland development were investigated using *in vivo* and *in vitro* approaches, including morphometric analysis of wholemounted mammary glands, quantification of histological markers, and primary mammary epithelial cell (MEC) functional and biochemical assays.

**Results:**

Analysis of Cdc42-overexpressing mammary glands revealed abnormal terminal end bud (TEB) morphologies, characterized by hyperbudding and trifurcation, and increased side branching within the ductal tree. Quantification of markers of proliferation and apoptosis suggested that these phenotypes were not due to increased cell proliferation or survival. Rather, Cdc42 overexpressing MECs were more migratory and contractile and formed dysmorphic, invasive acini in three-dimensional cultures. Cdc42 and RhoA activities, phosphorylated myosin light chain, and MAPK signaling, which contribute to migration and invasion, were markedly elevated in Cdc42 overexpressing MECs. Interestingly, Cdc42 overexpressing mammary glands displayed several features associated with altered epithelial-stromal interactions, which are known to regulate branching morphogenesis. These included increased stromal thickness and collagen deposition, and stromal cells isolated from Cdc42 overexpressing mammary glands exhibited elevated mRNA expression of extracellular matrix proteins and remodeling enzymes.

**Conclusions:**

These data suggest that Cdc42 overexpression disrupts mammary gland branching morphogenesis by altering Rho GTPase and MAPK signaling, leading to increased MEC contractility and migration in association with stromal alterations. Our studies provide insight into how aberrant Cdc42 expression may contribute to mammary tumorigenesis.

## Introduction

Cell division cycle 42 (Cdc42) is a member of the Ras homolog (Rho) family of small guanine nucleotide triphophatases (GTPases) that is overexpressed and hyperactivated in human breast invasive ductal carcinomas [1-3]. A number of studies in other cell and tissue types have demonstrated that Cdc42 regulates cell cycle progression, polarity, migration, cell fate determination, and differentiation, which are essential for mammary gland development and become disrupted during tumor formation [4-6]. We previously showed that Cdc42 is required for primary mammary epithelial cell (MEC) morphogenesis *in vitro* and that it regulates polarity establishment, proliferation, and survival of MECs during acinar morphogenesis [7]. Studies investigating the importance of Cdc42 during postnatal mammary gland development *in vivo* are currently ongoing in our laboratory, and our data indicate that Cdc42 knockout MECs are outcompeted by wild-type neighboring MECs (Bray and Vargo-Gogola, unpublished results). Thus, these loss-of-function studies indicate that Cdc42 is important for normal mammary gland morphogenesis *in vitro* and *in vivo*. However, our understanding of the mechanisms by which aberrant expression of Cdc42 disrupts mammary gland morphogenesis and facilitates tumor formation and progression *in vivo* has been limited until now due to a paucity of *in vivo* mouse models of Cdc42 overexpression and hyperactivation.

Postnatal mammary gland development is initiated in mice at 3 weeks of age when rising levels of ovarian hormones and local growth factors stimulate MEC proliferation and motility within terminal end buds (TEBs). The concerted action of MEC proliferation and motility drives TEB invasion and branching into the mammary fat pad, giving rise to an arborized ductal tree [8]. Mammary gland branching morphogenesis also requires reiterative interactions between the epithelial and stromal compartments, and both secreted factors and mechanical signals between the two compartments contribute to branch formation and patterning [9-11].

Rho family GTPases function within epithelia to integrate and transduce bidirectional soluble and mechanical signals between the epithelial and stromal compartments [12]. Studies indicate that Rho signaling coordinates cell proliferation and motility with changes in cell shape and intracellular contractility that are important for outgrowth and patterning of the branched mammary ductal tree. For example, real-time imaging studies using an *in vitro* model of mammary gland branching have revealed that Ras-related C3 botulinum toxin substrate 1 (Rac) and actomyosin contractility are required for mammary gland branch formation, whereas Rho-associated coiled-coil containing protein kinase (ROCK) functions to suppress hyperbranching and promote reorganization of the bilayered mammary epithelium [9,13]. Although Rac and ROCK have been implicated in mammary gland branching morphogenesis, the contribution of Cdc42 is not well understood.

To investigate the effects of Cdc42 overexpression during normal and neoplastic mammary gland development *in vivo* we developed a Cdc42 conditional overexpression mouse model and examined the effects of Cdc42 overexpression during postnatal mammary gland morphogenesis. We show that Cdc42 overexpression disrupts TEB morphogenesis and induces hyperbranching. Intriguingly, Cdc42 overexpression does not impact cell cycle progression to drive increased branching. Rather, it enhances MEC contractility and migration potentially by activating mitogen-activated protein kinase (MAPK) signaling. Our studies also suggest that Cdc42 overexpression in the mammary epithelium alters epithelial-stromal interactions, leading to stromal alterations that likely contribute to the epithelial phenotypes.

## Methods

### Mouse husbandry and care

Mice were housed in the University of Notre Dame Freimann Life Science Center. All studies were approved by the Institutional Animal Care and Use Committee at the University of Notre Dame and Indiana University School of Medicine (Protocol Number 14-015) and were conducted in accordance with the guidelines of the US Public Health Service Policy for Humane Care and Use of Laboratory Animals. All efforts were made to minimize suffering of the mice. Mice were fed a conventional diet or doxycyline (dox) containing chow *ad libitum* and were maintained at 21 to 22°C with a 12-hour light and 12-hour dark cycle. To induce transgene expression or control for any effects of dox, bitransgenic and control female mice were fed dox-containing chow (2 g/kg) (Bio-Serv Inc, Frenchtown, NJ, USA, S3893) for the specified number of weeks beginning at 4.5 weeks of age.

### Transgenic mice

To generate the tetracycline (tet)-regulatable Cdc42 transgenic mouse model we created the following construct. The approximately 600 bp wild-type human Cdc42 cDNA was subcloned from the pCMV-Sport6 vector (Thermo Scientific Open BioSystems, Waltham, MA, USA) using PCR. Primers used to create and subclone the insert were as follows: Forward-5′-CCG GAA TTC ATG TAC CCA TAC GAT GTT CCA GAT TAC GCT CAG ACA ATT AAG TGT GTT GTT GTG GGC GAT G-3′ and reverse-5′-CCG GAA TTC GAT GTT CAT AGC AGC ACA CAC CTG-3′. The forward primer contained an EcoRI site and an ATG start site. The reverse primer contained an EcoRI restriction site. The PCR product was gel purified and digested with EcoRI. The Cdc42 insert was then ligated into the TMILA tetracycline operator (TetO)-IRES-luciferase vector downstream of the TetO [14]. The construct was sequenced and then tested in MCF7 Tet-On cells [15], which confirmed inducibility. A 5.2 Kb fragment containing the TetO-Cdc42-IRES-luciferase cassette was microinjected into the pronuclei of fertilized FVB/N oocytes by the Transgenic and Knockout Mouse Core at the Indiana University School of Medicine, Indianapolis, IN, USA. PCR was used to identify founder mice, which were bred with mouse mammary tumor virus-reverse tetracycline transactivator mice (MMTV-rtTA) [16] already in use in our laboratory [17]. Mice were maintained on an inbred FVB/N background.

### Genotyping

Primer pairs used to genotype mice for the presence of the MMTV-rtTA transgene were: 5′-TCC AAG GGC ATC GGT AAA CA-3′ and 5′-GCA TCA AGT CGC TAA AGA AG-3′. Reaction conditions were 94°C for 3 min, followed by 30 cycles of 94°C for 30 s, 60°C for 45 s, and 72°C for 45 s followed by one cycle of 72°C for 5 min. Glycerol, 3 μl of a 50% solution, was added as part of a 25 μl total reaction volume to enhance product yield. Primer pairs used to genotype mice for the presence of the TetO-Cdc42 transgene were: 5′-CGT CAG ATC GCC TGG AGA CG-3′ and 5′-GAT GTT CAT AGC AGC ACA CAC CTG CGG-3′. Reaction conditions were 94°C for 3 min, followed by 30 cycles of 94°C for 1 min, 55°C for 30 s, and 72°C for 30 s, followed by one cycle of 72°C for 5 min.

### Wholemount mammary gland preparation and morphometric analysis

Number 4 left mammary glands were fixed immediately after dissection in 4% PFA in PBS for 2 h while rocking on ice. Glands were rinsed in PBS and stained in carmine overnight with gentle rocking followed by destaining and dehydration in a series of ethanols for 1 h each with gentle rocking. Glands were cleared in xylenes overnight and stored long term in methyl salicylate. A StereoImager (Carl Zeiss, Inc, Oberkochen, Germany) was used to image the carmine-stained glands at low magnification (16×) for quantification of gland size and at high magnification (80×) for quantification of branching, ductal dilation, and TEB morphology. Branching was quantified by counting the total number branch nodes found in three 4 × 10^6^ μm^2^ defined areas adjacent to the sides and leading edge of the lymph node. Ductal tree area was measured using the outline measurement tool in AxioVision 4.6 software (Zeiss). Ductal dilation was quantified using high magnification images (80×) and scoring the number of independent dilated regions found within the ductal tree from the back of the lymph node toward the leading end of the fat pad. Abnormal TEBs were defined as TEBs that were trifurcated or had multiple buds on the neck.

### Histological methods and image quantification

Glands were fixed immediately after dissection in 4% PFA in PBS for 2 h while rocking on ice, rinsed in PBS, and stored in 70% ethanol at 4°C until paraffin embedding. Five-μm sections were cut, deparaffinized in xylenes, and rehydrated. Antigen retrieval was performed by boiling the sections for 20 min in 10 mM sodium citrate buffer. Sections were blocked and antibodies were diluted in 5% BSA/Tween or M.O.M.™ block reagent and M.O.M.™ antibody diluents (Vector Laboratories, Burlingame, CA, USA, BMK2202) for mouse-derived primary antibodies. Sections were incubated with blocking buffer for 1 h, with primary antibodies overnight, and secondary antibodies for 1 h. Elite ABC Reagent (Vector Laboratories, PK7100) and DAB (Vector Laboratories, SK-4100) were used to develop staining for immunohistochemistry (IHC) and the slides were counterstained with hematoxylin (Thermo Fisher Scientific, CS400-1D). For immunofluorescence staining Alexa Fluor™ 488 anti-mouse (Molecular Probes, Eugene, OR, USA, A11001), Alexa Fluor™ 488 anti-rat (A11006), Alexa Fluor™ 555 anti-rabbit (A21428), and Texas Red™-X anti-rabbit (T-6391) secondary antibodies were used. Masson’s Trichrome staining to detect collagen was done and quantified as previously described [18]. At least five animals per group and a minimum of three TEBs per animal were analyzed for each experiment. Images were taken using an AxioImager (Zeiss). Images were quantified using the ImageJ cell counter plugin.

### Bromodeoxyuridine (BrdU) injection of mice

Control and Cdc42-overexpressing mice treated with dox-containing chow for three weeks were given intraperitoneal injections of 3 mg/ml BrdU (Sigma-Aldrich, St Louis, MO, USA, B-5002) in saline at 10 μl per gram bodyweight 2 h prior to euthanasia and dissection of the mammary glands. The glands were fixed and stained as described above.

### Organoid and fibroblast isolation for luciferase assays, GLISA, and qRT-PCR

Four-and-a-half-week-old Cdc42-overexpressing and control mice were treated with the dox diet for 1 week prior to euthanasia and mammary gland dissection. The 2, 3, and 4 mammary gland pairs were dissected, and lymph nodes were removed from the number 4 glands. Organoids and fibroblasts were isolated as previously described [18]. Briefly, the glands were manually minced and incubated in DMEM/F-12 (Thermo Fisher Scientific, SH30272) with 2 mg/ml collagenase A (Roche, Geneva, Switzerland, 10103578001), 100 units/ml hyaluronidase (Sigma-Aldrich, H3506), and 1 x antibiotic-antimycotic (Invitrogen, Carlsbad, CA, USA, 15240-062) for 1 h at 37°C with 200 rpm rotation at a 45° angle. The tissues were shaken manually at 30 min and 60 min during the digestion to aid in breaking apart the tissues. The cells were washed with DMEM/F-12 and centrifuged twice at 450 g for 10 min. The cells were incubated at room temperature for 3 min with manual shaking in DMEM/F-12 with 2 units/ml DNase I (Sigma-Aldrich, D2463) and centrifuged at 450 g for 10 min. Differential centrifugation was used to separate fibroblasts from organoids, which consisted of pulse centrifugation to 450 g with the supernatant from the first spin containing the fibroblasts. Fibroblasts and organoids for luciferase assays were immediately frozen. Fibroblasts for qRT-PCR were frozen in Trizol (Invitrogen, 15596-018) for RNA isolation.

### Single mammary epithelial cell isolation

Primary MECs used in *in vitro* assays were isolated from the 2, 3, and 4 mammary glands from mice treated for 1 week with dox. Initial steps for single cell isolation were identical to those used for organoid isolation as described above. Cells were washed in PBS and then digested in 0.05% trypsin + EDTA in PBS for 5 min at 37°C with 200 rpm rotation. An equivalent volume of wash buffer was added and cells were triturated at least 50 times with a p1000 pipette. Cells were spun at 600 g for 3 min. Cells were counted and used immediately for *in vitro* experiments or were frozen in 90% FBS + 10% DMSO freezing media and later thawed for some of the *in vitro* experiments.

### Luciferase assays

Lysates used for luciferase assay were prepped from pulverized frozen whole glands, organoids, or fibroblasts in passive lysis buffer (Promega, Madison, WI, USA, E1941). After a 10-min incubation on ice, the lysates were centrifuged at 13,000 rpm at 4°C for 10 min to remove debris. Lysates were allowed to warm to room temperature before luciferase substrate (Promega, E148) was added. A GloMax 20/20 Luminometer (Promega) was used to read luciferase activity. Values were normalized to total protein determined by BCA assay (Pierce, Rockford, IL, USA, 23225).

### Western blot analysis

Lysates used for western blot analysis were derived from whole glands snap frozen in liquid nitrogen immediately after dissection. Frozen glands were pulverized with a mortar and pestle followed by lysis in ice-cold RIPA buffer plus protease and phosphatase inhibitors (Pierce, 78440) for 10 min on ice. The lysates were then cleared by centrifugation at 10,000 rpm for 10 min at 4°C. BCA assay was used to determine lysate protein concentrations. Lysates were electrophoresed on 10% SDS-polyacrylamide gels and transferred onto PVDF membranes. Five percent milk in TBST was used for blocking and primary antibodies were diluted in 5% milk/TBST and incubated with the membrane for 2 h or overnight. Blots were probed with secondary HRP-conjugated antibodies (Jackson Immunologicals, West Grove, PA, USA) for 1 h. Phosphorylated primary antibodies were diluted in 5% BSA in TBST. Blots were developed using a GE Healthcare (Little Chalfont, UK) ImageQuant and ImageJ was used to calculate densitometry values.

### Antibody concentrations

The following antibodies were used at the indicated dilutions for the specified applications. Western analysis: β-actin 1:5000 (Sigma-Aldrich, A5441); Cdc42 1:1000 (BD Transduction Laboratories, San Jose, CA, USA, 610929); phosphorylated MLC ser19 1:1000 (Cell Signaling, Beverly, MA, USA, 3671); phosphorylated ERK 1:1000 (Cell Signaling, 4370); total Erk 1:1000 (Cell Signaling, 4695); phosphorylated p38 1:1000 (Cell Signaling, 4511); β-tubulin (Sigma-Aldrich, T5201). IHC/IF: Ki67 1:5000 (Abcam, Cambridge, UK, ab15580); BrdU 1:1000 (Thermo Fisher Scientific, MA3-071); CC3 1:1000 (Cell Signaling, 9661); phosphorylated histone-H3 1:5000 (Merck Millipore, Darmstadt, Germany, 06-570); F4/80 1:50, no antigen retrieval (Invitrogen, MF48000); phosphorylated ERM 1:1000 (Cell Signaling, 3141); E-cadherin 1:250 (BD Transduction, 610181); K14 1:400 (Covance, Leeds, UK, PRB-155P); K8 1:250 (Developmental Studies Hybridoma Bank, TROMA-I). The K8 monoclonal antibody developed by Philippe Brulet and Rolf Kemler was obtained from the Developmental Studies Hybridoma Bank developed under the auspices of the NICHD and maintained by The University of Iowa, Department of Biology, Iowa City, IA, USA, 52242.

### RNA isolation and qRT-PCR

RNA was isolated from control and Cdc42-associated fibroblasts from three mice per genotype pooled using Trizol and an RNeasy RNA purification column (Qiagen, Valencia, CA, USA, 91355) according to manufacturer’s recommendations. One μg of RNA was converted to cDNA using the RT^2^ First Strand Kit (Qiagen, 330401) and amplified using RT^2^ Profiler PCR Array Mouse Extracellular Matrix and Adhesion Molecules (PAMM-013A) per manufacturer’s instructions. Data were analyzed using the web-based software RT^2^ Profiler PCR Array Data Analysis from SABiosciences (Frederick, MD, USA). To validate gene expression changes identified by the array, cDNA was amplified using RT^2^ SYBR Green qPCR Master Mix (Qiagen, 330522), the StepOnePlus Real-Time PCR System (Applied Biosystems, Foster City, CA, USA), and the following primers: Col1a1 primers: 5′-GCT CCT CTT AGG GGC CAC T-3′ and 5′-CCA CGT CTC ACC ATT GGG G-3′; Fn1 primers: 5′-TTC AAG TGT GAT CCC CAT GAA G-3′ and 5′-CAG GTC TAC GGC AGT TGT CA-3′; Mmp2 primers: 5′-ACC TGA ACA CTT TCT ATG GCT G-3′ and 5′-CTT CCG CAT GGT CTC GAT G-3′; Mmp3 primers: 5′-ACA TGG AGA CTT TGT CCC TTT TG-3′ and 5′-TTG GCT GAG TGG TAG AGT CCC-3′; Mmp9 primers: 5′-CTG GAC AGC CAG ACA CTA AAG-3′ and 5′-CTC GCG GCA AGT CTT CAG AG-3′; and Gapdh primers: 5′-CCA ATG TGT CCG TCG TGG ATC-3′ and 5′-GTT GAA GTC GCA GGA GAC AAC-3′. The reaction was setup in triplicate and conditions were as follows: 95°C for 10 min then 40 cycles of 95°C for 15 s and 60°C for 1 min followed by a melting curve. Col1a1, Fn1, Mmp2, Mmp3, and Mmp9 mRNA levels were normalized to Gapdh mRNA levels and the data was analyzed using comparative C_T_.

### Cdc42, RhoA, and Rac1 activity assays on isolated organoids

GLISA Cdc42 Activated Assay Biochem Kit (Cytoskeleton, Denver, CO, USA, BK127), GLISA RhoA Activation Assay Biochem Kit (Cytoskeleton, BK121) and Rac1 Activation Assay Biochem Kit (Cytoskeleton, BK126) were used to measure levels of activated Cdc42, RhoA, and Rac1 according to the manufacturer’s instructions. Mammary organoid lysates were prepared using the kit lysis buffer. Organoids isolated from two to five mice were pooled per group after 1 week and 3 weeks of dox treatment and the assays were run in triplicate. All lysates were prepared within 10 min prior to snap freezing.

### Contraction assays

Primary MEC contractility was analyzed using the Cell Contraction Assay (Cell Biolabs, Inc., San Diego, CA, USA, CBA201) according to the manufacturer’s instructions. Growth media (MEGM BulletKit, Lonza, Walkersville, MD, USA, CC-3150) with 2 μg/ml dox was added once the gels solidified and changed when the gels were released and after each time point measurement. ROCK inhibitor, 25 μM Y27632 (Tocris Bioscience, Bristol, UK, 1254), or an equal volume of vehicle was added when the gels were released. Quantification of gel contraction was done using images of the gels taken immediately after their release and after 24 and 48 h post release to measure the difference in gel area from time of release. Imaging and quantification was done with a Zeiss Axioimager A1 epifluorescence microscope. Individual assays were conducted in duplicate or triplicate and averaged. Data without inhibitor are representative of four independent experiments and data with the ROCK inhibitor are representative of two independent experiments.

### *In vitro* migration assays

Cryopreserved primary MECs were used for these studies. Approximately 500,000 MECs were plated onto a 6-cm dish and allowed to adhere to the plate and form characteristic epithelial cobblestone patches in MEGM Bullet Kit Media + dox. The media was replaced with serum-free F12 + dox and the cells were serum starved for 24 h. The cells were washed with PBS, trypsinized with 0.05% trypsin for 15 min and removed. Cells were then spun at 600 g for 3 min and resuspended in F12 media + dox and plated onto 8 μm-pore transwell filters (BD Transduction Laboratories) into 24-well plates (75,000 cells per well). Eight hundred μl of serum containing MEGM media was added to each well below the filter. The cells were allowed to migrate through the filter for 24 h at which time the upper surface of the filter was scraped twice with a cotton swab and media was suctioned off to remove any cells that did not migrate through the filter. The filters were then fixed in 4% PFA for 20 min and permeabilized for 10 min with 0.05% Triton-X 100 (Thermo Fisher Scientific, BP151). The filters were then removed from the well, transferred to a glass slide, and mounted with Vectashield + DAPI (Vector Laboratories, H-1200). A minimum of nine, 200× fields per filter were quantified and the total number of migrated cells was recorded per experiment. The fold changes of total migrated cells between control and Cdc42-overexpressing MECs were averaged from four independent experiments. Seven control mice and 11 Cdc42 overexpressing mice are represented in the data.

### Three-dimensional (3D) culture assays

Primary MECs were isolated and plated on tissue culture plastic plates. MECs from at least three mice were pooled per group for each experiment. Plates were treated with 2% Matrigel containing MEGM media for at least 1 h at 37°C prior to plating of the cells. Cells were allowed to adhere to the plate and form characteristic epithelial cobblestone patches. After 48 to 72 h, the cells were washed with PBS, trypsinized with 0.05% trypsin for 15 min and removed. Cells were then spun at 600 g for 3 min and resuspended at 15,000 or 30,000 cells per well in 40 μl Matrigel per well of an 8-well chamber slide. The gel was allowed to solidify for 20 min at 37°C and 400 μl of warm MEGM + 2% Matrigel + 2 μg/ml dox was added to each well. The media was replaced every 3 days and the cultures were analyzed after 5 days using immunostaining and a Zeiss LSM 7 confocal microscope. Entire wells were quantified for each experiment. Invasive acini were defined as structures made up of five or more cells that had an invasive protrusion or at least one cell actively migrating away from the acinus. Data represent the average fold change between control and Cdc42-overexpressing MECs in three independent experiments. Dysmorphic acini were defined as acini with nonspherical morphologies with or without invasive protrusions or cells migrating away from the acinus. Data represent the average fold change between control and Cdc42 overexpressing acini in a total of three wells per group from three independent experiments.

For the spindle orientation three-dimensional culture assays, cryopreserved primary MECs were used and plated as described above. After 48 h, the cultures were fixed, immunostained with antibodies to tubulin and α6-integrin to identify the spindle and basal surface, respectively, and quantified using confocal microscopy. Acini were defined as structures with three or more clustered cells as previously described [7], and the first 25 acini identified with a mitotic spindle were quantified. Data represent the percentages of structures with the spindle parallel, perpendicular, or angled relative to the basal surface of the forming acinus out of the total number of structures. Two independent experiments were performed and MECs from at least four mice were pooled per group for each experiment.

### Three-dimensional culture immunofluorescence (IF) staining

Staining was performed using methods adapted from Debnath *et al*., [19]. Three-dimensional acini were fixed with 2% or 4% PFA for 20 min at room temperature, permeabilized with 0.5% Triton-X 100 in PBS for 10 min, washed in 7.5 mg/ml glycine (Thermo Fisher Scientific, G46-1) in PBS. IF buffer consisted of 7.7 mM NaN3, 0.1% BSA, 0.2% Triton X-100, and 0.05% Tween-20 in PBS. Invasive three-dimensional culture assay wells were stained with Alexa Fluor™ 488 phalloidin (Molecular Probes, Carlsbad, CA, USA, A12379) diluted 1:50 in IF buffer for 1 h and TO-PRO3 (Molecular Probes, T3605) diluted 1:200 in PBS for 10 to 20 min. Mitotic spindle orientation culture wells were stained overnight with α6-integrin (Millipore, MAB1378) diluted 1:200 in IF buffer + 10% goat serum (Sigma-Aldrich, G9023) and α-tubulin (Abcam, Ab1825) diluted 1:400 in IF buffer + 10% goat serum. Wells were stained with Alexa Fluor™ 488 goat anti-rat (Invitrogen, A11006) and Alexa Fluor™ 555 goat anti-rabbit (Molecular Probes, A21428) secondary antibodies diluted 1:200 in IF buffer + 10% goat serum for 1 h and TO-PRO3 diluted 1:200 in PBS for 10 to 20 min. All slides were mounted with 4 μl Vectashield + DAPI per well with coverslips and allowed to dry in the dark for 24 to 72 h before sealing coverslips with nail polish and imaging.

### Flow cytometry analysis

Single MECs isolated as described above were suspended in 1 ml of PBS and fixed by adding 2.5 ml of 100% ethanol. Ethanol was added 500 μl at a time while gently vortexing to prevent clumping, and cells were fixed on ice for 15 min and stored at 4°C until analysis. Cells were pelleted by centrifugation at 600 g for 5 min and resuspended in propidium iodide (PI) staining solution (50 μg/ml PI; 0.1 mg/ml RNAse A; 0.05% Triton X-100) and incubated for 30 min in a 37°C water bath. The cells were transferred using a 26 G syringe through a cell strainer cap of a flow tube (BD Falcon, Franklin Lakes, NJ, USA, 352235) to break up clumps. At least 10,000 events were analyzed using a Beckman Coulter (Brea, CA, USA) FC500 Flow Analyzer for PI fluorescence intensity. MECs from two to three mice were pooled for each experiment. Data are representative of two independent experiments.

### Statistical analysis

Unpaired Student’s *t* test was used for all statistical tests. *P* values less than 0.05 were considered significant. Error bars represent the standard error of the mean.

## Results

### Generation of TetO-Cdc42-overexpressing mice

**Figure 1:**
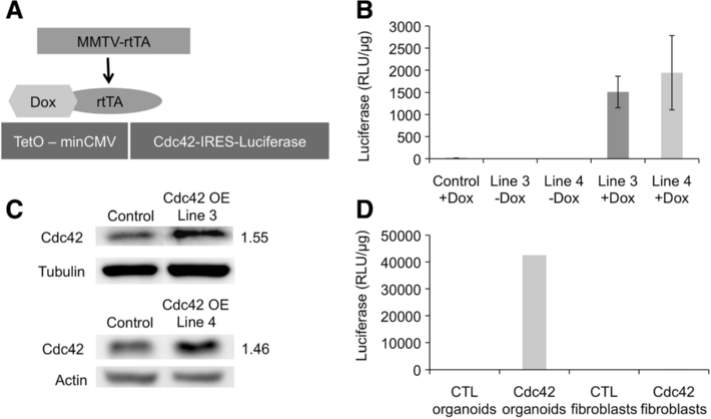
**Generation of the conditional Cdc42 overexpression mouse mammary gland model. (A)** Schematic model of TetO-Cdc42/MMTV-rtTA bigenic mice. The TetO-Cdc42 transgene contains the tetracycline operator and minimal cytomegalovirus (CMV) promoter with human Cdc42 and IRES luciferase immediately downstream. TetO-Cdc42 mice were bred with the MMTV-rtTA mice to drive transgene expression in the mammary gland [16]. In the presence of dox, rtTA activates expression of the Cdc42 transgene. Dox-treated MMTV-rtTA mice serve as controls for all studies. **(B)** Graph depicts average luciferase levels of control (MMTV-rtTA, + dox) and lines 3 and 4 bigenic (TetO-Cdc42/MMTV-rtTA, -/+ dox) mammary glands (n = 15,1,1,10,14). Transgene is induced only in bigenic mice treated with dox. **(C)** Western blot analysis of Cdc42 expression on five pooled whole gland lysates per group after 3 weeks (Line 3) and 1 week (Line 4) of dox treatment. Densitometry values are normalized to loading control and displayed as fold change compared to control. **(D)** Graph shows luciferase levels of mammary organoids and stromal cells isolated from Cdc42 overexpressing and control mice (n = 3 animals pooled per group). Cdc42, cell division cycle 42; dox, doxycycline; MMTV-rtTA, mouse mammary tumor virus reverse tetracycline transactivator; TetO, tetracycline operator.

To investigate the effects of Cdc42 overexpression on the developing mammary gland we generated a regulatable Cdc42 overexpression mouse model. In this model, overexpression of wild-type Cdc42 is induced in the mammary gland by feeding TetO-Cdc42/MMTV-rtTA bitransgenic mice (referred to as Cdc42-overexpressing (OE) mice) doxycycline (dox)-containing chow. To create these mice the wild-type human Cdc42 cDNA was inserted into a TetO-IRES-luciferase construct [14], verified by sequencing, and tested for functionality using MCF-7 Tet-On breast cancer cells [20] (Figure 1A and data not shown). Pronuclear injection of the construct yielded 42 potential founder mice, and screening for the presence of the transgene by PCR led to the identification of five positive lines. All five lines were bred to the MMTV-rtTA mice, which express the rtTA in the TEBs and ducts of the developing postnatal mammary gland [16]. Beginning at 4.5 weeks of age, TetO-Cdc42/MMTV-rtTA and MMTV-rtTA control mice were fed either dox or non-dox-containing chow to determine which lines were inducible as well as the levels of Cdc42 overexpression. After 1 week, the mice were euthanized, and mammary glands were dissected for analysis. Whole mammary gland lysates were prepared and luciferase assays were done to rapidly screen for transgene expression. Four of the five founder lines expressed the luciferase transgene at levels approximately 10 to 100 fold over controls in an inducible fashion. Two of the lines, designated lines 3 and 4 (L3 and L4), were chosen for further analysis because they expressed similar levels of luciferase activity (Figure 1B). Western blot analysis of whole gland lysates showed that Cdc42 protein levels were increased approximately 1.5 fold in both lines after 1 or 3 weeks of dox treatment compared to dox-treated MMTV-rtTA control mice (Figure 1C).

To confirm that the transgene was confined to the epithelial compartment, stromal cells and mammary organoids were isolated from control and line 4 mammary glands after 1 week of dox treatment. Our methods for stromal cell isolation yield a relatively pure population with approximately 0.25% MEC contamination based on immunostaining for markers of epithelial and stromal cells (data not shown) [18]. Consistent with our analysis of whole mammary gland lysates, a high level of luciferase activity was detected in the mammary organoids from line 4 mice, but not in control organoids or stromal cells from either control or Cdc42-overexpressing mammary glands (Figure 1D). Combined, these data show that the Cdc42 transgene can be inducibly overexpressed in the mammary glands of two independent transgenic lines and that expression of the transgene is restricted to the mammary epithelium.

### Cdc42 overexpression in the developing mammary gland induces abnormal TEBs and hyperbranching of the ductal tree

**Figure 2:**
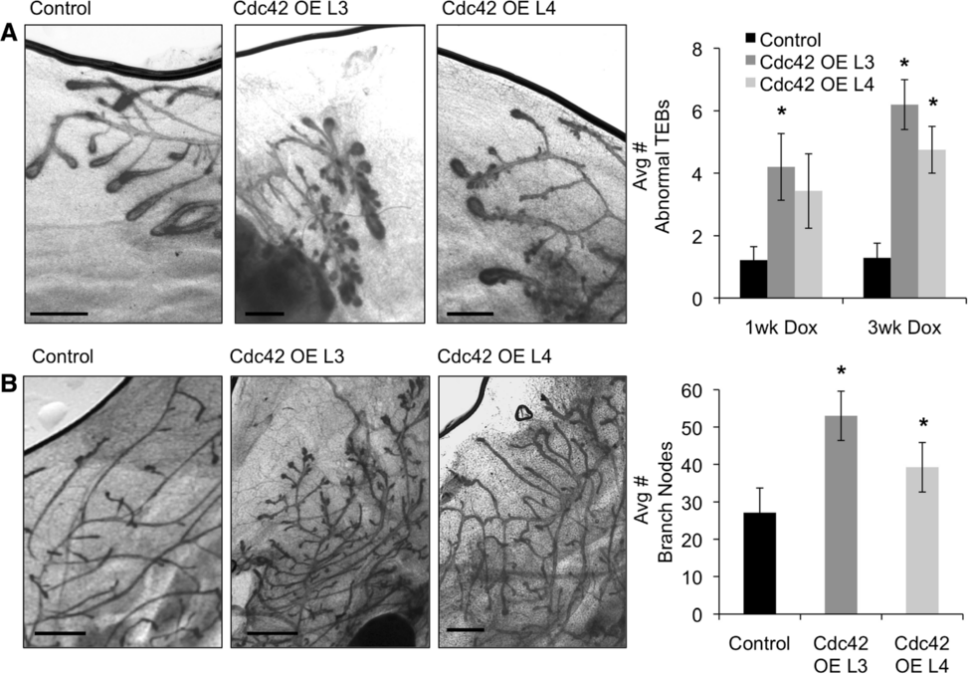
**Cdc42-overexpressing mammary glands display abnormal terminal end bud (TEB) morphologies and increased side branching. (A)** Representative images of wholemounted mammary glands from 1 week dox-treated line 3, line 4, and control mice. Scale bar = 1000 μm. Graph depicts average number of abnormal TEBs (TEBs with multiple hyperbuds or trifurcations) per gland after 1 week or 3 weeks of dox treatment in lines 3 and 4 compared to dox-treated control mice (1 week n = 9,5,7 *P* = 0.01,0.07; 3 weeks n = 7,5,4 *P =<0.01). **(B)** Representative images of whole mounted mammary glands from 5-week dox-treated mice. Scale bar = 1000 μm. Graph depicts average number of branch nodes after 5 weeks of dox treatment in three 4 × 10^6^ μm^2^ regions of the ductal tree on sides and leading edge of lymph node in number 4 mammary glands (line 3 n = 7,4 *P = 0.005; line 4 n = 8, *P = 0.001; control n = 7).

We examined the effects of continuous Cdc42 overexpression at early (5.5 weeks of age), middle (7.5 weeks of age), and late (9 weeks of age) time points in the developing postnatal mammary gland. Analysis of whole mounted mammary glands at 5.5 and 7.5 weeks of age, after 1 and 3 weeks of transgene expression, respectively, revealed that Cdc42 overexpression induced abnormal TEB morphologies characterized by hyperbudding and trifurcation in both lines 3 and 4 (Figure 2A). The increased presence of hyperbudded and trifurcated TEBs suggested that long-term Cdc42 overexpression would lead to increased branching of the ductal tree. Quantification of branch points in whole mounted mammary glands at 9 weeks of age, the developmental time point when postnatal mammary gland development is typically complete in the FVB/n strain of mice, showed a significant increase in side branching in the mammary glands of lines 3 and 4 as compared to mammary glands from dox-treated control mice (Figure 2B). Additional defects were noted in the Cdc42-overexpressing mammary glands, including a mild reduction in total ductal tree area, persistence of TEBs at the late developmental time point, and regions of ductal dilation (Additional file 1). The increased ductal branching was the most remarkable phenotype present in the Cdc42-overexpressing mammary glands, and we chose to pursue studies to define the mechanisms underlying this phenotype.

**Figure 3:**
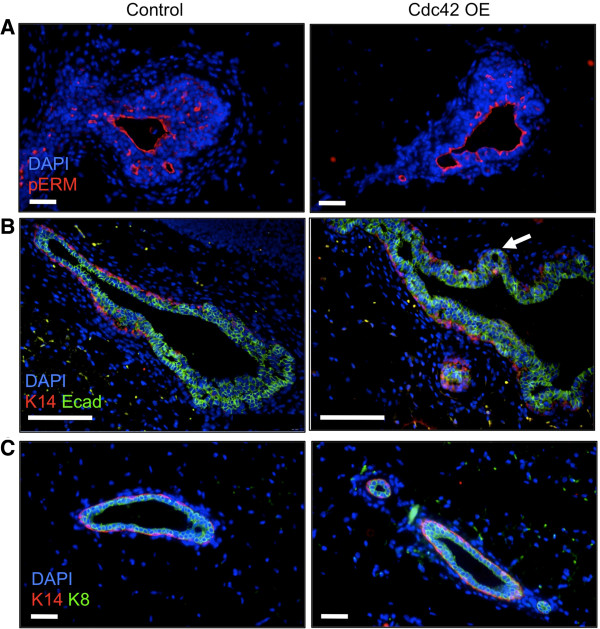
**Cdc42-overexpressing mammary glands do not display defects in apical or basal-lateral polarity or development of the myoepithelial or luminal cell compartments. (A)** Representative images of TEBs from control and Cdc42-overexpressing mice immunostained to detect the apical surface marker phosphorylated ERM (n = 5 animals per genotype with at least three TEBs analyzed per animal). Scale bars = 20 μm. **(B)** Representative TEBs immunostained using antibodies against E-cadherin and keratin 14 to visualize the body and myoepithelial cell compartments, respectively (n = 5 animals per genotype with at least three TEBs analyzed per animal). E-cadherin marks the surface of the body cells. Arrow indicates gap in myoepithelial layer at a bud forming off a large TEB. Scale bars = 50 μm. **(C)** Representative images of mature ducts immunostained to detect keratins 8 and 14 (n = 5 animals per genotype). Scale bars = 20 μm. Cdc42, cell division cycle 42; ERM, ezrin-radixin-moesin; TEB, terminal end bud.

To begin to investigate the mechanisms that might be contributing to the hyperbudded TEB and branching phenotypes, we examined whether Cdc42 overexpression was affecting apical and basal-lateral polarity establishment or development of the myoepithelial and luminal cell compartments. Immunostaining to detect the apical surface marker phosphorylated ezrin-radixin-moesin (pERM) and the basal-lateral surface marker E-cadherin was done on mammary gland tissue sections from dox-treated mice. No differences were detected in the localization or intensity of either marker within the TEBs or ducts, suggesting that Cdc42 overexpression does not disrupt the establishment of apical or basal-lateral polarity (Figure 3A-B). We also performed immunostaining to detect the myoepithelial cell marker keratin 14 (K14). K14-positive myoepithelial cells localize to the neck region, whereas the K14-negative cap cells localize to the middle and tip regions of the TEBs. We noted that gaps in the K14-positive myoepithelial layer were detectable at sites where branches were forming, and gaps were more frequent in the Cdc42-overexpressing TEBs (Figure 3B). These results are consistent with published studies showing that myoepithelial cells actively migrate and partially cover growing branches [9], which are more abundant in the Cdc42-overexpressing mammary glands. Gaps in the myoepithelial layer were rarely detected in fully formed ducts (Figure 3C). Collectively, these data indicate that Cdc42 overexpression does not result in obvious defects in polarity establishment or development of the myoepithelial and luminal compartments.

### Cdc42 overexpression does not affect mammary epithelial cell proliferation or survival rates

**Figure 4:**
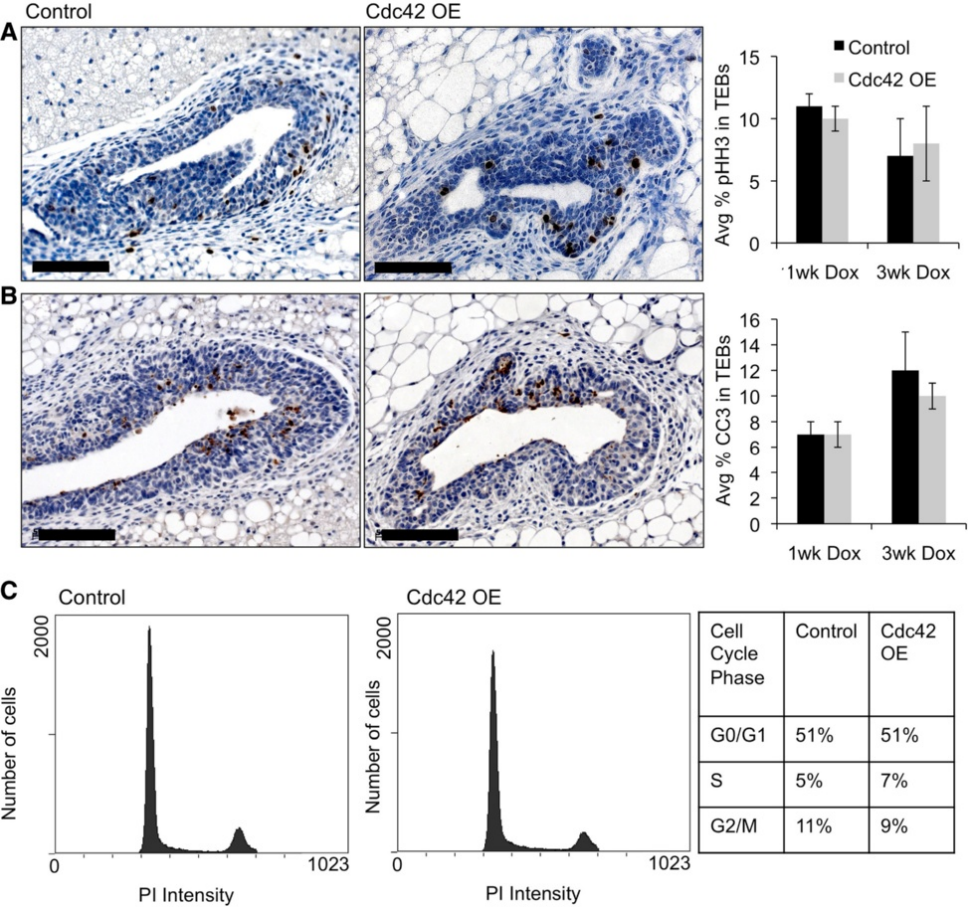
**MEC proliferation and survival rates are not altered in Cdc42-overexpressing mammary glands. (A)** Representative images of pHH3 immunostaining on tissue sections from line 4 and control mammary glands treated for 1 week with dox. Scale bar = 50 μm. Graph shows average percentage of pHH3-positive cells ± SEM in TEBs after 1 week and 3 weeks of dox treatment (n = 3,3;3,4, *P* = 0.43;0.77). **(B)** Representative images of CC3 immunostaining on tissue sections from line 4 and control mammary glands treated for 1 week with dox. Scale bar = 50 μm. Graph depicts average percentage of CC3-positive cells ± SEM in TEBs after 1 week and 3 weeks of dox treatment *in vivo* (n = 5,5;3,4 *P* = 0.53;0.59). **(C)** Histograms represent flow cytometry analysis of isolated primary control and Cdc42-OE MECs stained with propidium iodide (PI) after 1 week of dox treatment *in vivo.* Table shows the percentage of cells in each phase of the cell cycle out of total live cells (≥67,000 events) demonstrating that control and Cdc42 OE MECs have similar cell cycle profiles. Data are representative of two independent experiments (n = 5 mice per genotype). CC3, cleaved caspase-3; Cdc42, cell division cycle 42; MEC, mammary epithelial cell; OE, overexpressing; pHH3, phosphorylated histone-H3; TEB, terminal end bud.

Branching of the mammary gland ductal tree is dependent on cell proliferation [9], and we previously demonstrated that Cdc42 is a crucial regulator of MEC proliferation [7]. We therefore hypothesized that Cdc42 overexpression may increase proliferation rates to drive hyperbranching. To investigate the effects of Cdc42 overexpression on MEC proliferation, mammary gland tissue sections from 5.5- and 7.5-week-old dox-treated mice were immunostained for proliferation markers, including a mitosis marker phosphorylated histone-H3 (pHH3), an active cell cycle marker Ki67, and an S phase marker bromodeoxyuridine (BrdU) incorporation (Figure 4A and Additional file 2). Interestingly, quantification of these markers did not reveal any differences in proliferation rates between Cdc42-overexpressing and control TEBs or ducts at either time point. Immunostaining and quantification of cleaved caspase 3 in the TEBs was done to evaluate whether Cdc42 overexpression might be increasing MEC survival, and again, no differences in apoptosis rates were detected between the Cdc42-overexpressing and control mammary glands (Figure 4B). To further confirm these results the percentages of MECs in each phase of the cell cycle were analyzed by performing flow cytometry on freshly isolated, PI-stained MECs from mice treated for 1 week with dox (Figure 4C). Indeed no differences in the cell cycle profiles were detected between the two groups. These results suggest that the hyperbudded TEBs and increased side-branching phenotypes that were present in the Cdc42-overexpressing mammary glands were not due to defects in cell cycle progression of the MECs.

### Cdc42 overexpression enhances MEC migration and invasion

**Figure 5:**
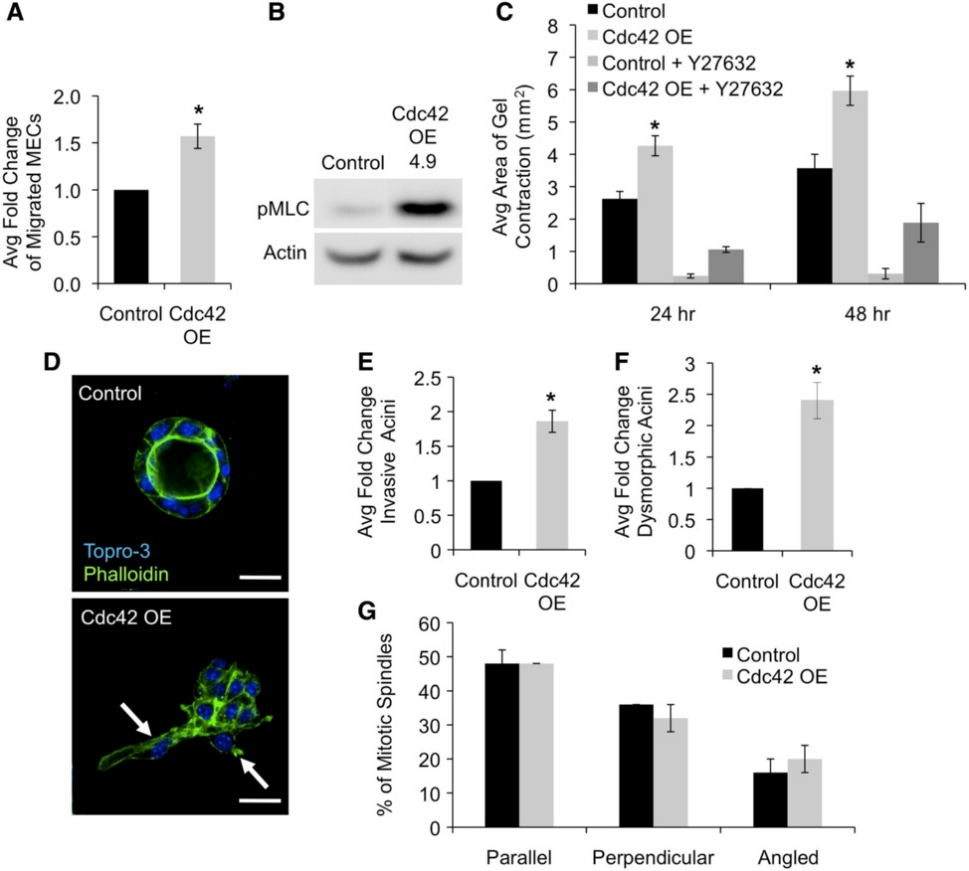
**Cdc42 overexpression enhances MEC migration and contractility and development of dysmorphic, invasive mammary acini. (A)** Transwell migration assay analysis of primary MECs from line 4 and control mammary glands treated for 1 week with dox prior to isolation. Fold change (± SEM) of total cells migrated was quantified per experiment. Data represent average of four independent experiments (n = 7 control animals; n = 11 Cdc42 OE mice, *P = 0.005). **(B)** Western blot analysis of phosphorylated MLC on lysates prepared from whole mammary glands after 1 week of dox treatment (n = 5 animals pooled per group). Densitometry was normalized to actin loading control and is represented as fold increase compared to control. **(C)** Graph depicts average area of collagen gel contraction (± SEM) at 24 and 48 h post release with and without the ROCK inhibitor Y27632, (*P = 0.03). Data are representative of four independent experiments. **(D)** Three-dimensional culture morphogenesis assay of primary MECs from 1-week dox-treated line 4 and control mammary glands. Representative confocal images depict control and abnormal Cdc42-overexpressing acini. Arrows indicate invasive protrusions. Scale bar = 25 μm. **(E)** Graph depicts average fold change (± SEM) of total invasive acini per well from three independent experiments (*P = 0.007). **(F)** Graph shows the average fold change in dysmorphic acini per well from three independent experiments (*P = 0.008). **(G)** Analysis of mitotic spindle orientation in line 4 and control mammary acini (n = 50 spindles per genotype analyzed in two independent experiments). Cdc42, cell division cycle 42; MEC, mammary epithelial cell; MLC, myosin light chain; OE, overexpressing.

In addition to proliferation, cell migration is another critical mechanism that contributes to mammary gland branch formation [9]. Cell migration involves a multistep process that requires both cell contraction and forward movement and is known to be regulated by Rho signaling [21,22]. Because Cdc42 overexpression did not impact cell cycle progression, we reasoned that the increased branching could be due to enhanced cell migration. To investigate this, we measured the ability of Cdc42-overexpressing MECs to migrate using a transwell assay. Primary MECs were serum starved, plated in serum-free medium in the upper chamber of the transwell, and serum-containing medium was added to the bottom chamber to establish a concentration gradient. The MECs were allowed to migrate for 24 h and then fixed to prevent subsequent cell division. Quantification of the number of migrated MECs showed that Cdc42-overexpressing MECs were significantly more migratory compared to control MECs (Figure 5A).

Actomyosin contractility contributes to cell migration, and we were interested in determining whether Cdc42-overexpressing MECs were also more contractile. Western blotting showed that phosphorylated myosin light chain (MLC) was markedly upregulated in the Cdc42-overexpressing mammary glands (Figure 5B). Next, we examined the contractility of the Cdc42-overexpressing MECs using a collagen gel contractility assay in which MECs were plated in a collagen gel and the gel was released from the plate after 48 h. At the time of release, the cultures were treated with the ROCK inhibitor Y27632, which is activated by Rho GTPases and regulates MLC-mediated contractility [23]. Analysis of the change in gel area after 24 and 48 h showed that the Cdc42-overexpressing MECs were significantly more contractile than control MECs and that ROCK inhibition blocked MEC contractility, confirming the importance of Rho GTPase-mediated actomyosin contractility in this process (Figure 5C).

The increased contractility and motility of the Cdc42-overexpressing MECs suggested that they would also be more invasive. We therefore investigated whether Cdc42 overexpression would promote invasion of MECs undergoing morphogenesis in three-dimensional Matrigel cultures. Single MECs were seeded in Matrigel, and after 6 days the cultures were stained with fluorescent-tagged phalloidin and analyzed using confocal microscopy. Cdc42 overexpression resulted in a significant increase in the number of invasive acini, which were defined as acini with an invasive protrusion or at least one cell migrating away from the acinus (Figure 5D-E). A significant increase in the presence of dysmorphic acini, which were defined as elongated, flattened, or nonspherical acini, was also detected in the Cdc42-overexpressing cultures (Figure 5D-F). These data are consistent with our *in vivo* results demonstrating aberrant TEB morphologies and increased branching in Cdc42-overexpressing mammary glands.

Cdc42 affects epithelial organization in part through regulation of mitotic spindle orientation [24]. We considered the possibility that Cdc42 overexpression might be altering spindle orientation to promote the formation of dysmorphic and invasive acini. To investigate this, Cdc42 overexpressing and control acini were stained with α-tubulin and α6-integrin to visualize the mitotic spindles and basal surface of the acini, respectively. Spindle orientation was scored as parallel, perpendicular, or angled with respect to the basal surface. This analysis showed that Cdc42 overexpression does not alter spindle orientation in developing acini (Figure 5G), suggesting that spindle orientation defects do not contribute to the formation of the abnormal Cdc42-overexpressing acini.

**Figure 6:**
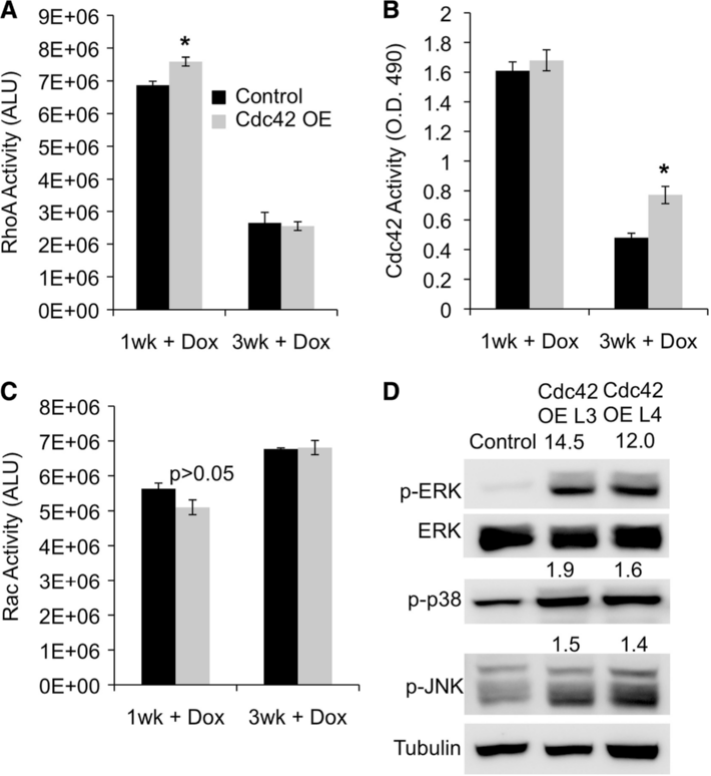
**Cdc42 and RhoA activities and MAPK signaling are upregulated in Cdc42-overexpressing mammary glands. (A)** Graph depicts RhoA activity as average light units (ALU) (± SEM) in lysates prepared from mammary organoids isolated from line 4 and control mice after 1 week and 3 weeks of dox treatment (*P = 0.02). Data represent four animals at 1 week and two animals at 3 weeks per group. **(B)** Graph shows Cdc42 activity as average OD 490 readings (± SEM) from lysates prepared from organoids isolated from line 4 and control mice after 1 week and 3 weeks of dox treatment (*P = 0.01). Data represent four animals per group at 1 week and three animals per group at 3 weeks. **(C)** Rac activity assay results are representative of three animals per group at 1 week and five animals per group at 3 weeks. **(D)** Western analysis of phosphorylated ERK, P38, and JNK in lysates prepared from whole mammary glands after 3 weeks of dox treatment (n = 5 pooled animals per group). Densitometry values are normalized to tubulin loading control and represented as fold change compared to control. Cdc42, cell division cycle 42; ERK, extracellular signal-related kinase; JNK, c-Jun N-terminal kinase; MAPK, mitogen-activated protein kinase; Rho, Ras homolog gene family, member A.

Elevated Rho GTPase activity and downstream activation of MAPK signaling has been shown to increase MEC contractility, disrupt MEC morphogenesis, and promote invasion [25,26]. To determine whether deregulated Rho GTPase activity and MAPK signaling might contribute to the Cdc42 overexpression phenotypes, we investigated the effects of Cdc42 overexpression on Rho GTPase activity in the developing mammary epithelium. Organoids were isolated from 1- and 3-week dox-treated mice, and Cdc42, RhoA, and Rac activities were quantified using GLISA assays. Interestingly, a small, but significant increase in RhoA activity was detected after 1 week of dox treatment in Cdc42-overexpressing MECs relative to control MECs (Figure 6A). In contrast, Cdc42 activity was not altered at this time point. After 3 weeks of dox treatment, however, Cdc42 activity was significantly increased in Cdc42-overexpressing MECs compared to control MECs, whereas RhoA activity was no longer elevated (Figure 6A-B). No changes in Rac activity levels were detected at either time point (Figure 6C). To determine if MAPK signaling was also altered in the Cdc42-overexpressing mammary glands we performed western blotting for phosphorylated MAPK proteins on mammary gland lysates prepared from lines 3 and 4 and control mice, which showed a marked increase in phosphorylated extracellular signal-related kinase (ERK), p38, and c-Jun N-terminal kinase (JNK) (Figure 6D). These data indicate that Cdc42 overexpression results in dynamic regulation of RhoA and Cdc42 activities and increased MAPK activity in the developing mammary epithelium, which likely contribute to the Cdc42 overexpression-mediated MEC phenotypes *in vitro* and *in vivo*.

### Cdc42-overexpressing mammary glands exhibit features associated with stromal activation

**Figure 7:**
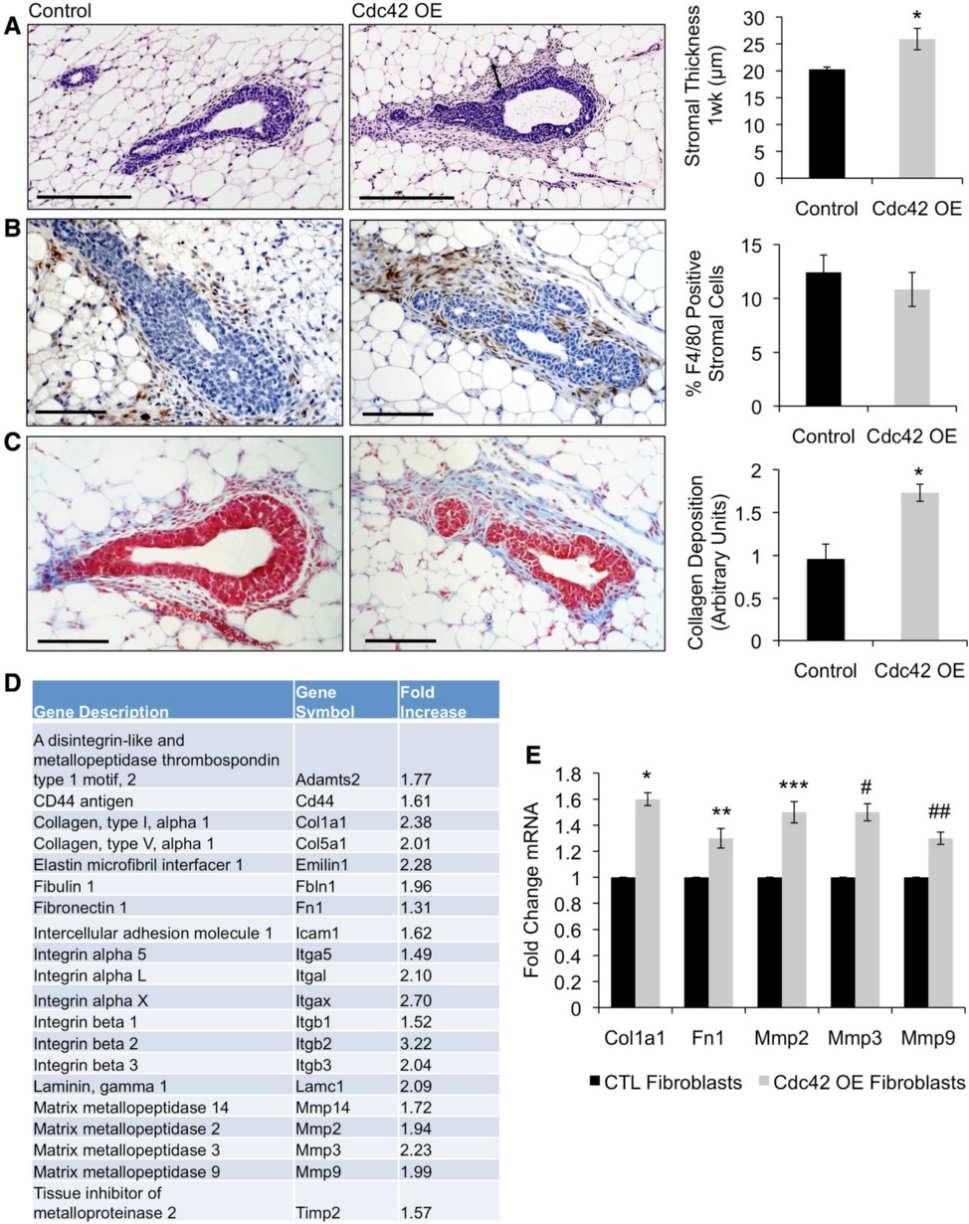
**Cdc42-overexpressing mammary glands display characteristics associated with stromal activation. (A)** Representative images of H&E-stained longitudinally sectioned TEBs from 1 week dox-treated Cdc42-overexpressing and control mammary glands. Scale bar = 100 μm. Graph depicts average stromal thickness (± SEM) at neck region of TEBs (n = 7,5; *P = 0.008). **(B)** Representative images of F4/80-immunostained TEBs from 1 week dox-treated Cdc42-overexpressing and control mammary glands (n = 4 to 5 animals per genotype, with at least three TEBs analyzed per mouse). Scale bar = 50 μm. Graph shows percentage of F4/80-positive stromal cells (± SEM). **(C)** Representative images of Masson’s trichrome-stained TEBs (blue) (n = 4 animals per genotype, with an average of three TEBs analyzed per mouse). Scale bar = 50 μm. Graph depicts quantification of collagen deposition (*P= 0.009). **(D)** Table of qRT-PCR Super Array results showing genes that were upregulated at least 1.5 fold in stromal cells isolated from Cdc42-overexpressing compared to control mammary glands. Data are representative of three animals per genotype. **(E)** qRT-PCR and new primer sets were used to validate several genes identified in the Super Array. Graph shows fold change in mRNA expression levels (*P = 0.0003, **P = 0.04, ***P = 0.003, ^#^*P* = 0.001, ^##^*P* = 0.005). Data are representative of three animals per group. Cdc42, cell division cycle 42; TEB, terminal end bud.

Crosstalk between the epithelial and stromal compartments is known to play an important role in normal and neoplastic mammary gland development. More specifically, extracellular matrix (ECM) deposition and remodeling by stromal cells contributes to mammary gland branching morphogenesis and patterning of the ductal tree [27], and aberrant ECM deposition and remodeling disrupts MEC morphogenesis and facilitates invasion [25,26,28,29]. Previously, we reported that abnormal TEB morphogenesis and hyperbranching of the ductal tree occurred in p190B RhoGAP-overexpressing mice in association with increased ECM deposition [17]. We were therefore interested in determining if ECM deposition was altered in the mammary glands of the Cdc42-overexpressing mice. First, we measured the thickness of the stroma in the neck region adjacent to the TEBs in H&E-stained tissue sections. This analysis demonstrated that the stroma associated with the Cdc42-overexpressing TEBs was significantly thicker in comparison to control TEBs (Figure 7A). To determine if expansion of the stromal cell population contributed to the increased stromal thickness, cell proliferation in the stroma adjacent to the TEBs was quantified using Ki67 staining. However, no differences in proliferation rates were detected, suggesting that expansion of the stromal population did not account for the increased ECM deposition (data not shown). We also performed F4/80 immunostaining to analyze macrophage infiltration, which is important for TEB and branching morphogenesis [30]. Furthermore, increased macrophage infiltration has been shown to promote mammary gland hyperbranching [31]. Quantification of F4/80+ cells did not reveal any differences in the number of infiltrating macrophages associated with the Cdc42 overexpressing compared to control TEBs (Figure 7B). However, further analysis will be required to determine whether changes in the activation status of the macrophage population contribute to the Cdc42-overexpressing mammary gland phenotypes.

To evaluate the composition of the thickened ECM, tissue sections were stained with Masson’s trichrome, and collagen accumulation in the stroma adjacent to the TEBs was quantified. These data show that increased collagen deposition contributed to the stromal thickness surrounding the Cdc42-overexpressing TEBs (Figure 7C). To further investigate the stromal changes, we performed qRT-PCR Super Array analysis for adhesion and ECM genes on cDNA prepared from freshly isolated stromal cells and found that mRNA expression levels for several integrin, collagen, and matrix metalloprotease (MMP) family members were upregulated greater than 1.5 fold in the stromal cells from Cdc42-overexpressing compared to control mammary glands (Figure 7D). qRT-PCR using additional primer sets was done to validate five of these genes, which confirmed that mRNA expression levels of type I collagen, fibronectin, MMP-2, MMP-3, and MMP-9 were elevated in the stromal cells from Cdc42-overexpressing mammary glands (Figure 7E). Collectively, these data suggest that overexpression of Cdc42 in the mammary epithelium alters epithelial-stromal interactions, leading to increased ECM deposition and potentially ECM remodeling.

## Discussion

Cdc42 is overexpressed and hyperactivated in human breast tumors [2,3]. However, the effects of Cdc42 overexpression on mammary gland development and the stochastic process of tumor formation *in vivo* have not been previously investigated due to a lack of Cdc42-overexpressing mouse models. Here we describe the generation of a novel conditional Cdc42-overexpressing mouse model and the effects of Cdc42 overexpression during postnatal mammary gland development. We show that overexpression of Cdc42 disrupts TEB morphogenesis and promotes hyperbranching of the ductal tree in association with stromal alterations. Furthermore, our data suggest that increased MEC contractility and migration, potentially via upregulation of MAPK signaling, contributes to these phenotypes.

Although Cdc42 is overexpressed and hyperactivated in breast tumors [2,3], mutations in Cdc42 have not been found [32]. Thus, regulation of Cdc42 protein translation, stability, and GTP hydrolysis and exchange rates likely underlie Cdc42’s altered expression and activity in tumors. When we designed the TetO-Cdc42 model, we aimed to accurately recapitulate this regulation, and therefore, chose to overexpress wild-type Cdc42 rather than a constitutively active mutant. Using this approach, we showed that Cdc42 and RhoA activities are differentially elevated in the mammary epithelium of the Cdc42-overexpressing mice during the developmental time course that we examined.

Cdc42 is upregulated early in MECs undergoing morphogenesis [33], and it is essential for polarity establishment and proliferation during the early stages of MEC morphogenesis [7,34,35]. These studies together with our data demonstrating that Cdc42 overexpression disrupts MEC acinus morphology in three-dimensional cultures and TEB architecture *in vivo*, suggest that precise regulation of Cdc42 activity is important for proper MEC morphogenesis and branching. Normal MECs likely have multiple mechanisms to ensure that proper levels of Cdc42 activity are maintained. One potential mechanism could involve RhoGDI1, which functions to sequester and maintain Rho GTPases in their inactive state in the cytoplasm [36]. Levels of RhoGDI1 are limited, and overexpression of one Rho GTPase can displace another family member, resulting in increased activation of the displaced family member [37]. This mechanism could account for the absence of elevated Cdc42 activity and concomitant increase in RhoA that we observed at the early developmental time. At the mid-developmental time point after the early stages of MEC morphogenesis and initiation of branching are complete, precise control of Cdc42 activity may no longer be crucial and elevated Cdc42 activity may facilitate branch elongation by enhancing MEC migration and invasion. Increased stromal deposition affects Rho GTPase activity in MECs [26,38], and it is possible that the stromal alterations detected in the Cdc42-overexpressing mammary glands also impact RhoA and Cdc42 activation. Further studies will be required to define the mechanisms regulating Rho GTPase activity in normal and transformed MECs.

It is interesting to note that overexpression of Cdc42 and p190B RhoGAP in the developing postnatal mammary gland have similar phenotypes, including abnormal TEBs, hyperbranching, and increased stromal deposition [17]. Similar to the Cdc42-overexpressing MECs, p190B-overexpressing MECs also display increased contractility [18]. Thus, disruption of the Rho signaling network in MECs, leading to increased intracellular contractility may play an important role in driving these phenotypes. Indeed, actomyosin-mediated contractility regulates mammary organoid branching *in vitro*[9].

One distinction between the p190B- and Cdc42-overexpressing mammary gland phenotypes is that p190B-overexpressing MECs exhibited both increased migration and proliferation [17,39], whereas Cdc42-overexpressing MECs displayed only increased migration. We anticipated that Cdc42 overexpression might enhance proliferation as our published studies investigating the effects of Cdc42 knockout during primary MEC morphogenesis *in vitro* demonstrated that Cdc42 is a critical regulator of MEC proliferation [7]. Furthermore, mammary gland branching is driven by both MEC proliferation and migration [9]. However, our studies suggest that Cdc42 overexpression facilitates aberrant branching morphogenesis by promoting increased MEC contractility and migration in the absence of any effects on proliferation.

The promigratory effects of Cdc42 overexpression in MECs are supported by the literature describing other Cdc42 gain of function models. A migration phenotype was reported in mouse embryonic fibroblasts isolated from Cdc42GAP knockout mice in which elevated Cdc42 activity disrupted directional migration [40]. In addition, neutrophils isolated from the Cdc42GAP knockout mice had an increased ability to migrate, but the direction of migration was disrupted [41]. Interestingly, MAPK signaling contributed to the migration phenotype in the Cdc42GAP knockout neutrophils, which showed changes in ERK and p38 phosphorylation that were similar to those detected in the Cdc42-overexpressing mammary glands. MAP kinases, including ERK, p38, and JNK, have been broadly implicated as regulators of cell proliferation and migration in diverse cell types in response to a variety of stimuli [42-44]. Our detailed analysis of cell cycle progression and apoptosis in the Cdc42-overexpressing mammary glands did not reveal any alterations in cell proliferation or survival. Thus, elevated MAPK activity in the Cdc42-overexpressing mammary glands may regulate MEC migration and invasion to promote hyperbranching.

Disruption of epithelial architecture is an important hallmark of breast cancer initiation, it contributes to invasion and metastasis, and it can be used to help predict survival [45-48]. The abnormal TEB morphologies detected in the Cdc42-overexpressing mammary glands together with our reported loss-of-function studies demonstrating the requirement for Cdc42 during the early stages of MEC acinus formation [7], suggest that Cdc42 is a key regulator of mammary epithelial architecture. Thus, Cdc42 overexpression may cooperate with initiating oncogenes to facilitate the disruption of epithelial architecture during the early stages of tumorigenesis. The increased migratory and invasive capacity of the Cdc42-overexpressing MECs suggests that Cdc42 overexpression may facilitate mammary tumor cell invasion and metastasis *in vivo*, and indeed, studies investigating the effects of Cdc42 knockdown in breast cancer xenografts have shown that Cdc42 regulates tumor cell invasion and metastasis *in vivo*[49]. Furthermore, an intriguing possibility is that Cdc42 overexpression may function during the early stages of tumor formation to induce protumorigenic and proinvasive stromal alterations. Future studies will be aimed at using this novel mouse model to determine the contribution of Cdc42 during different stages of tumor formation and progression and to define the molecular mechanisms by which aberrant Cdc42 expression facilitates these processes. A better understanding of how Cdc42 overexpression impacts the development and progression of breast cancer will help to pinpoint when targeting Cdc42 would be most effective and will define how best to target its aberrant actions.

## Conclusions

Overexpression of Cdc42 in the developing mammary gland induces aberrant TEB morphogenesis and hyperbranching of the ductal tree in association with stromal alterations. Elevated MAPK signaling leading to increased MEC contractility and migration likely contributes to the Cdc42-overexpressing mammary gland morphogenetic defects. This novel mouse model will serve as an important tool to define the cellular and molecular mechanisms by which Cdc42 overexpression affects mammary tumor formation, progression, and metastasis *in vivo*.

## Abbreviations

BCA: Bicinchoninic acid assay; BrdU: 5-bromo-2-deoxyuridine; BSA: Bovine serum albumin; Cdc42: Cell division cycle 42; DMEM/F-12: Dulbecco’s modified Eagle’s medium nutrient mixture F-12; dox: Doxycycline; ECM: Extracellular matrix; ERK: Extracellular signal-related kinase; GAP: GTPase-activating protein; GEF: Guanine nucleotide exchange factor; GTPase: Guanine nucleotide triphophatase; IF: Immunofluorescence; IHC: Immunohistochemistry; JNK: c-Jun N-terminal kinase; L3: Transgenic founder line number 3; L4: Transgenic founder line number 4; MAPK: Mitogen-activated protein kinase; MEC: Mammary epithelial cell; MEGM: Mammary epithelial cell growth medium; MLC: Myosin light chain; MMP: Matrix metalloprotease; MMTV-rtTA: Mouse mammary tumor virus reverse tetracycline transactivator; OE: Overexpression; PBS: Phosphate-buffered saline; PI: Propidium iodide; qRT-PCR: Quantitative reverse-transcription polymerase chain reaction; Rac: Ras-related C3 botulinum toxin substrate 1; RhoA: Ras homolog gene family, member A; ROCK: Rho-associated coiled-coil containing protein kinase; TEB: Terminal end bud; TetO: Tetracycline operator.

## Competing interests

The authors declare no competing interests.

## Authors’ contributions

KB designed and performed experiments and analyzed data, including analysis of Cdc42 expression and signaling, mammary gland phenotypes, immunostaining, and three-dimensional culture experiments. KB also helped draft the manuscript. MG performed contraction assays, immunostaining, q-RT-PCR, and analyzed data. JY performed migration assays, GLISA assays, spindle analysis, analyzed mammary gland phenotypes, and analyzed and interpreted results. EL performed and analyzed immunostaining on tissue sections. MH and JCS generated and tested the construct to make the TetO-Cdc42-overexpressing mice. MH performed initial characterization to identify inducible Cdc42-overexpressing transgenic lines. TVG conceived of the study and designed and directed the experiments, analyzed data, and drafted the manuscript. All authors have read and approved the final manuscript.

## Supplementary Material

Additional file 1 (A) Whole mount mammary gland images and quantification of average number of terminal end buds (TEBs) in animals with TEBs (≥100 μm diameter) (± SEM) in 5-week dox-treated whole mounts (n = 8,9; *P = 0.03). (B) Whole mount mammary gland images and average number of dilated regions (± SEM) per whole mount (from back of lymph node toward leading edge of ductal tree) (n = 9,9; *P <0.02). (C) Whole mount images and average ductal tree area (± SEM) of 5-week dox-treated animals (n = 8,9; *P <0.02). Click here for file

Additional file 2 (A) Average percentage of Ki67-positive cells (± SEM) in terminal end buds (TEBs) after 1 week and 3 weeks of dox treatment *in vivo *(n = 9,8;3,4 *P = 0.34;0.88). (B) Average percentage of BrdU-positive cells (± SEM) in TEBs after 3 weeks of dox treatment *in vivo* (n = 3,4; *P = 0.23). BrdU, 5-bromo-2-deoxyuridine. Click here for file
