# Supplementary figures and images for: Cdc42 overexpression induces hyperbranching in the developing mammary gland by enhancing cell migration
Source: Breast Cancer Res. 2013 Sep 30;15(5):R91. doi: 10.1186/bcr3487 (PMC3978759; doi:10.1186/bcr3487)

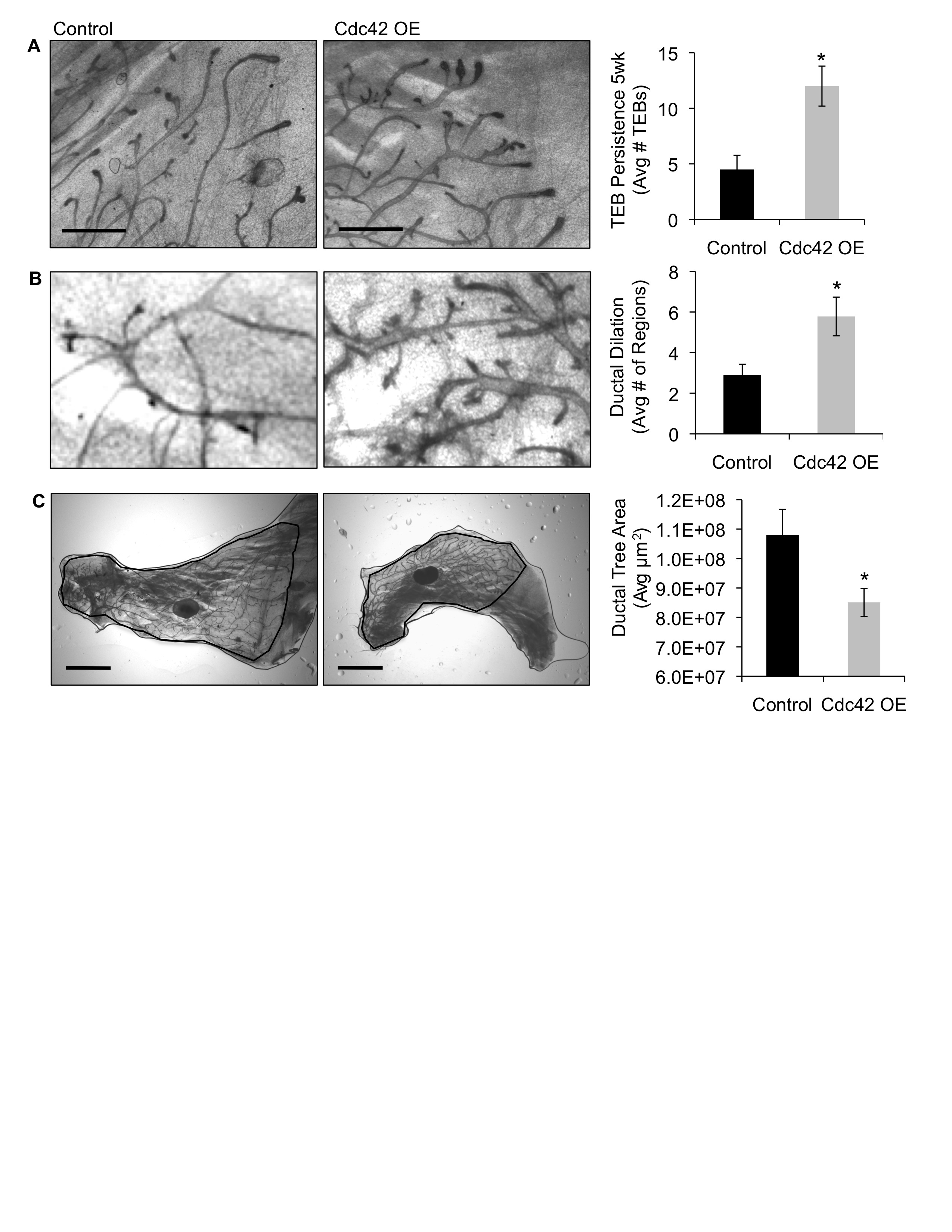

Supplement: Additional file 1 — (A) Whole mount mammary gland images and quantification of average number of terminal end buds (TEBs) in animals with TEBs (≥100 μm diameter) (± SEM) in 5-week dox-treated whole mounts (n = 8,9; *P = 0.03). (B) Whole mount mammary gland images and average number of dilated regions (± SEM) per whole mount (from back of lymph node toward leading edge of ductal tree) (n = 9,9; *P <0.02). (C) Whole mount images and average ductal tree area (± SEM) of 5-week dox-treated animals (n = 8,9; *P <0.02). [file bcr3487-S1.jpeg]

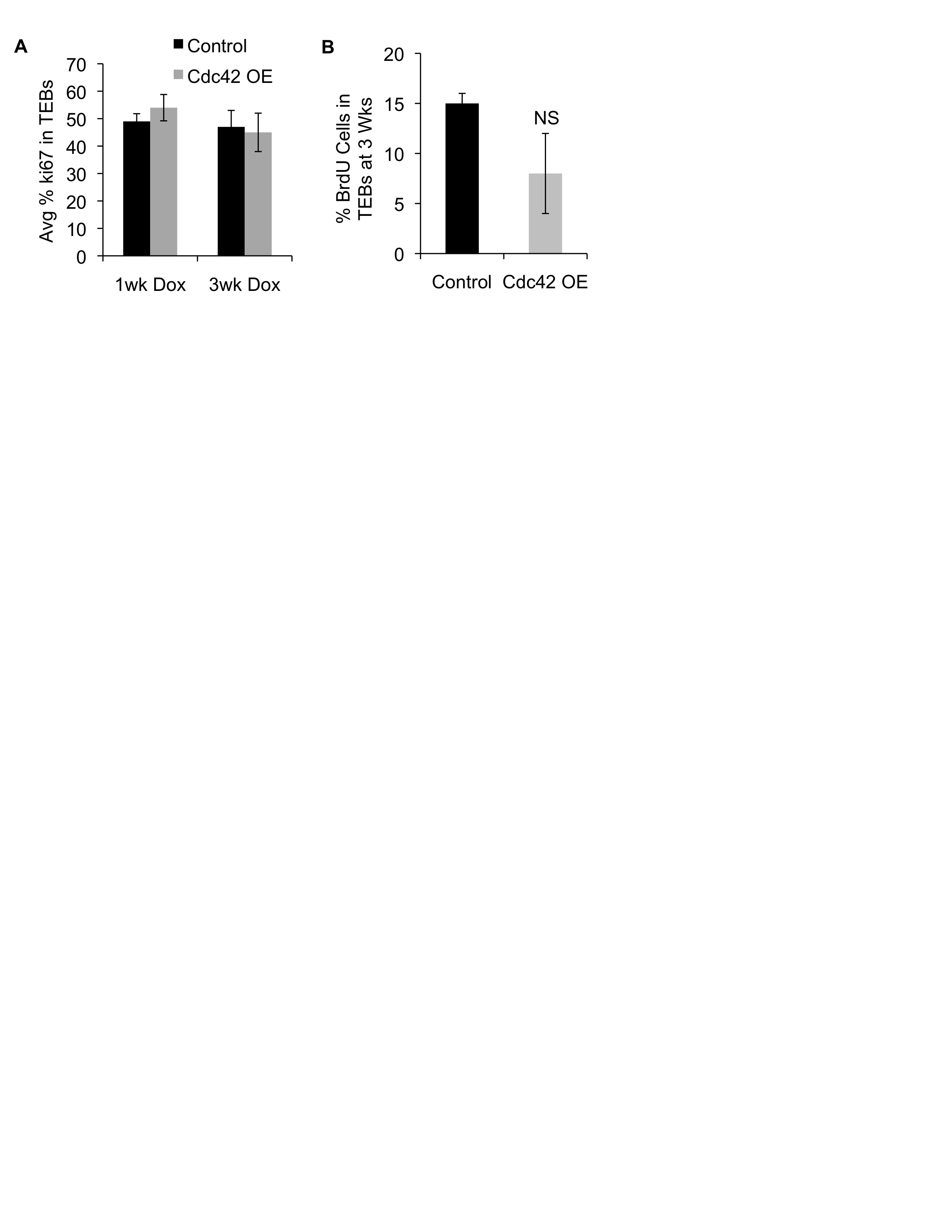

Supplement: Additional file 2 — (A) Average percentage of Ki67-positive cells (± SEM) in terminal end buds (TEBs) after 1 week and 3 weeks of dox treatment in vivo (n = 9,8;3,4 *P = 0.34;0.88). (B) Average percentage of BrdU-positive cells (± SEM) in TEBs after 3 weeks of dox treatment in vivo (n = 3,4; *P = 0.23). BrdU, 5-bromo-2-deoxyuridine. [file bcr3487-S2.jpeg]
